# Impact on refractive surgery due to increasing use of personal protection equipment: Insights from EUROCOVCAT group

**DOI:** 10.1177/11206721211018641

**Published:** 2021-05-27

**Authors:** Arthur B Cummings, Cian Gildea, Antoine P Brézin, Boris E Malyugin, Ozlem Evren Kemer, Omid Kermani, Isabel Prieto, Robert Rejdak, Miguel A Teus, Daniele Tognetto, Sandrine Zweifel, Mario D Toro

**Affiliations:** 1 Wellington Eye Clinic, Dublin, Ireland; 2 Université de Paris, Hôpital Cochin, Paris, France; 3 S. Fyodorov Eye Microsurgery Federal State Institution, Moscow, Russia; 4 University of Health Sciences, Ankara City Hospital, Ankara, Turkey; 5 Augenklinik am Neumarkt Schildergasse, Köln, Germany; 6 Department of Ophthalmology, Fernando Fonseca Hospital, Amadora, Portugal; 7 Chair and Department of General and Pediatric Ophthalmology, Medical University of Lublin, Lublin, Poland; 8 Department of Ophthalmology, University of Alcalá, Madrid, Spain; 9 Eye Clinic, Department of Medicine, Surgery and Health Sciences, University of Trieste, Trieste, Italy; 10 Department of Ophthalmology, University Hospital of Zürich, University of Zürich, Zürich, Switzerland; 11 Faculty of Medical Sciences, Collegium Medicum, Cardinal Stefan Wyszyński University, Warsaw, Poland

**Keywords:** Mask wearing, fogging spectacles, refractive surgery, LASIK, refractive lens exchange, cataract surgery, COVID-19 pandemic

## Abstract

Since the World Health Organization declared COVID-19 to be a pandemic on 11th March 2020, changes to social and sanitary practices have included significant issues in access and management of eye care during the COVID-19 pandemic. Additionally, the fear of loss, coupled with social distancing, lockdown, economic instability, and uncertainty, have led to a significant psychosocial impact that will have to be addressed. In the current COVID-19 pandemic, personal protective equipment such as face masks or face coverings have become a daily necessity. While “mass masking” along with hand hygiene and social distancing became more widespread, new issues began to emerge – particularly in those who wore spectacles as a means of vision correction. As we began to see routine patients again after the first lockdown had been lifted, many patients visited our clinics for refractive surgery consultations with a primary motivating factor of wanting spectacle independence due to the fogging of their spectacles as a result of wearing a mask. In this article, we report on new emerging issues in eye care due to the widespread use of masks and on the new unmet need in the corneal and cataract refractive surgery fields.

## Introduction

The impact of the COVID-19 pandemic has been far reaching to say the least. Initial reactions were primarily focused on the effects of the disease – its morbidity, virulence and how to avoid contracting it.^1^ As time progressed, society began to understand that the effects of this virus were much farther reaching than initially thought.^2^ The societal and economic impacts of lockdowns were obvious but as we continued to navigate the path of the disease, people began reporting more of the day-to-day challenges of living with this virus.^3–5^ The fear of loss, coupled with social distancing, lockdown, economic instability, and uncertainty, have led to a significant psychosocial impact that will have to be addressed. In this scenario, personal protective equipment such as face masks or face coverings have become a daily necessity, more than a simple recommendation not only among the health care practitioners but also in all routine activities during the COVID-19 pandemic.^6,7,8^

The European COVID-19 Cataract Group (EUROCOVCAT) is a group of ophthalmic surgeons and experts, currently from 11 countries. We have organized conference calls to share our different experiences and perspectives on the current situation. In previous publications, we have already highlighted the impact of COVID-19 outbreak on ophthalmological care in different health care systems, its future consequences in terms of disability, and access to sight-saving cures for many patients.^9–11^ In this article, we report on new emerging issues in eye care due to the widespread use of masks and on the new unmet need in the corneal and cataract refractive surgery fields.

## Discussion

From an eye care practitioner viewpoint, the timeline of the virus manifested in reports of viral conjunctivitis to increased risk of contracting the disease, due to the close contact nature of our work.^11^ A logical finding published by The Lancet, showed that front line healthcare workers were most at risk of contracting COVID-19.^12^ Of these medical professionals, the three subspecialties at highest risk were anesthesiologists, emergency medicine physicians and ophthalmologists.^12,13,14^ The medical professional in China who first alerted people to this new disease and who later succumbed to the virus, was an ophthalmologist.^15^

However, as mask wearing became more widespread, new issues began to emerge^16^ particularly in those who wore spectacles as a means of vision correction.^17,18^ As we began to see routine patients again after lockdown, many people were presenting for refractive surgery consultations with a primary motivating factor of wanting spectacle independence due to fogging of their spectacles because of wearing a mask. In our clinics, unexpectedly, we had patients requesting to undergo refractive surgery due to mask-associated spectacle fogging. In general, we have noted a significant increase of around 25%versus the same period (average number collected from the EUROCOVCAT Group) in the previous year, in the numbers of patients presenting for refractive surgery at a time when we would have possibly expected the opposite to be a truer reflection of the reality we find ourselves in. Indeed, in literature for several ophthalmic diseases and ophthalmological treatments the data have shown a dramatic drop (references) in different departments.^19–21^ However, we believe that the reason is that the neovascular age-related macular degeneration (nAMD) patient population is usually significantly older and more vulnerable with regards to potential COVID-19 infection. The main reason for the drop is the fear of getting infected in a clinical setting. Similar effects are seen with cataract patients, also usually older aging 60–80 years.^11^ The patients and findings we report in our paper concern a completely different patient population. Indeed, refractive surgery patients are usually younger, aged 20–40 years. They are more mobile and are less fearful of COVID-19. Also, many continue to work even during lockdown. Thus, the refractive surgery patient population is significantly impacted by a side effect of the pandemic, namely the necessity to wear masks, that then leads to the fogging of spectacles lenses and the dehydration of contact lenses. This leads to frustration and forms the main trigger to start the process of exploring refractive surgery as a potential solution to their problems. We have also learned that some newer refractive surgery practices are less busy than those that have been around for longer and are better known in the community. A possible explanation may be that those in practice for longer and hence those that have a stronger word-of-mouth reputation have benefited more than the less experienced colleagues. Anecdotally, in discussion with other refractive surgeons, there is some consensus that patients are seeking out the more experienced surgeons and clinics now. There seems to be a renewed interest in personal health, but patients are preferring to see those with more experience and greater reputations even if the cost may be less at alternative clinics.

It is well known that the global demand for refractive surgical procedures such as laser refractive surgery, presbyopia-correcting surgery, refractive lens exchange (RLE), and phakic intraocular lens implantation (ICL), is expected to grow at a compound annual rate of 5.2% from 2018 to 2023. According to the latest estimates, this growth could increase up to 9.6% by 2025 with an annual surgical volume rising from 3.6 million to 5.8 million procedures.^22^ In 2018, the major markets included the US, Japan, and some Western European countries.^23^

Although economic conditions have improved in major markets in recent decades, progress in eyewear and contact lens technology, along with a shift in visual demand due to the prevalence of digital devices, has dampened the growing demand for refractive surgery in many developed countries. In the future, Market Scope expects a modest increase in demand for procedures based on technological advances, continuous economic improvement and rising rates of myopia in developed countries.^22^ In contradiction, after these series of pandemic lockdowns and consequently, in most cases, a new way of living, we have detected a significant increase in corneal refractive patients (mainly laser-assisted in situ keratomileusis – LASIK) and cataract refractive patients up to 25% versus the same period of time before pandemic (mainly RLE with premium IOLs) attending our private clinics. It is important to note that public institutions are also experiencing an increase in demand for the same reason.

We currently have limited data to predict the future behavior of the COVID-19 pandemic. Even though many governments have already started vaccination programs, it is predicted that this phase will last several months, and possibly even more than a year; and personal protection equipment (PPE) use will still be highly recommended or even mandatory until a significant percentage of the population has been vaccinated. Refractive surgery is an elective procedure, and it is indisputable that resumption of eye care services may not be effective immediately in certain areas.^24^ However, the new “mass masking” phenomenon is likely to continue to drive demand for refractive surgery due to spectacle fogging for the near future.

We looked at several methods to help glasses wearers overcome fogging issues, from anti-fog lens wipes to anti-fogging lens pastes, but no matter what we tried the issue persisted.^17^ On the other hand, we found in several cases the need to pause a refraction to clear a fogged phoropter lens, or to stop to wipe a condensed slit lamp ocular or an indirect ophthalmoscopy lens.^17,18^ These are real issues, not just for patients, but also for practitioners.

Another interesting finding associated with mask wearing is a new phenomenon coined as MADE, or Mask Associated Dry Eye.^25^ Both fogging of spectacles and MADE are a result of ill-fitting masks, particularly around the bridge of the nose. If there is a gap between the mask and the face, it will allow warm exhaled air to travel upwards over the eyes. It has been postulated that this increase in hot airflow over the ocular surface can increase evaporation rates and induce dryness, although the exact mechanism needs further study.^26^

Furthermore, a concern of which eye care practitioners need to be cognizant is the reporting of increased levels of post-operative infection. Both endophthalmitis in post-cataract surgery and LASIK flap infections have experienced a resurgence in recent months, the origins of which have been attributed to oral and nasopharyngeal flora,^27^ usually inconsequently expelled but now, being redirected upwards over the ocular surface due to the wearing of masks. It is something we should keep at the forefront of our minds when examining post-operative patients. We need to seal the upper border of the mask over the bridge of the nose during and after surgery so that exhaled breath is directed downwards to escape from the bottom edge of the mask.

Regarding contact lens wear, two main important issues need to be addressed. First, a recent study has shown that over time the risk of microbial keratitis in contact lens wearers, especially lenses with prolonged wearing time compared to LASIK is significantly higher.^28^ Secondly, contact lens wear often leads intolerance of contact lenses in the experience of refractive surgeons,^29^ as many patients seeking vision correction surgery have encountered an issue with their contact lenses. Thus, it is easy to suspect that an increased incidence of MADE^25^ during the ongoing COVID-19 pandemic may be an additional risk factor to develop a keratitis or increase the percentage of intolerance in contact lens wearers.

At the end of March 2020, Sugnak et al.^30^ show that the relevant ACE2 and TMPRSS2 proteins, to which the virus can dock, are most abundant in the middle nasal mucosa and not in the pharynx, as originally suspected. In addition, the same proteins were also detected in the human cornea. Colavita et al.^31^ reported that the virus in the conjunctival secretions for days and weeks, indicating that the tear film is not sufficiently virucidal to cope with SARS-CoV-2.

Publications from the U.S. National Institute of Health warned about the persistence of SARS-CoV-2 on carbon and silicone surfaces over a period of 3–5 days (https://www.nih.gov/news-events/news-releases/new-coronavirus-stable-hours-surfaces). Contact and smear infection are, in addition to droplets and aerosol the known routes of infection for COVID-19.^32^ Also, the American Academy of Ophthalmology (AAO) published a warning in March 2020 regarding careless handling of contact lenses on its website and recommended contact lenses wearers switch to glasses to stop spread of COVID-19. Indeed, contact lens wearers tend to touch their eyes and face more often than the average person.^33^ The potential risk of smear infection as a route of infection from the finger to the eye is highly likely, although such a case has not yet been reported. However, it is possible that a redundant contamination could lead to a gradual progression of infection if the contact lenses are contaminated viral load continuously is being transmitted via the nasolacrimal duct to the mid nasal mucosa. We urgently encourage scientific effort to clarify this question.

## Conclusion

The COVID-19 pandemic has brought to light many challenges, some expected, but many unexpected. It is how we react to these unexpected challenges which drives change and innovation, not just within our own field as eye care practitioners, but to society as a whole. Our patients are our primary concern; and to be able to support them by offering solutions to new problems, which arise in their lives, will always be the reward for our endeavors. Indeed, we cannot underestimate the role of PPE in our “new normal” lifestyle and its consequences, in particular, the trend of our patients’ needs to eliminate spectacles using refractive technologies to avoid spectacle fogging. Additionally, we need to take further steps to protect postoperative patients as well as contact lens wearers from infections due to the wearing of facemasks and coverings. For all these reasons, now more than ever, the preoperative discussion between the ophthalmologist and patient is of great importance to understand the reasons behind a refractive choice and to stress the pros/cons and specific risks due to COVID-19 and mask wearing.
